# Reply to Riva et al. Comment on “Gaspari et al. Blood Purification in Hepatic Dysfunction after Liver Transplant or Extensive Hepatectomy: Far from the Best-Case Scenarios. *J. Clin. Med.* 2024, *13*, 2853”

**DOI:** 10.3390/jcm14030822

**Published:** 2025-01-27

**Authors:** Rita Gaspari, Paola Aceto, Giorgia Spinazzola, Edoardo Piervincenzi, Maurizio Chioffi, Felice Giuliante, Massimo Antonelli, Alfonso Wolfango Avolio

**Affiliations:** 1 Department of Basic Biotechnological Science, Intensive Care and Peri-Operative Clinics, Università Cattolica del Sacro Cuore, 00168 Rome, Italy; rita.gaspari@unicatt.it (R.G.); massimo.antonelli@unicatt.it (M.A.); 2 Department of Emergency, Anesthesiological and Reanimation Sciences, Fondazione Policlinico Universitario Agostino Gemelli IRCCS, 00168 Rome, Italy; giorgia.spinazzola@policlinicogemelli.it (G.S.); edoardo.piervincenzi@policlinicogemelli.it (E.P.); maurizio.chioffi@gmail.com (M.C.); 3 Department of Gastroenterological, Endocrine, Metabolic and Nephro-Urological Sciences, General Surgery and Hepatobiliary Unit, Fondazione Policlinico Universitario Agostino Gemelli IRCCS, 00168 Rome, Italy; felice.giuliante@unicatt.it (F.G.); alfonsowolfango.avolio@unicatt.it (A.W.A.); 4 Department of Translational Medicine and Surgery, Università Cattolica del Sacro Cuore, 00168 Rome, Italy; 5 Department of Gastroenterological, Endocrine, Metabolic and Nephro-Urological Sciences, General Surgery and Transplantation Unit, Fondazione Policlinico Universitario Agostino Gemelli IRCCS, 00168 Rome, Italy

We sincerely appreciate the authors’ comments [1] on our study [2]. Their criticism has allowed us to better elucidate our experience with CytoSorb^®^ in patients with severe liver failure after liver transplantation or major liver resection. The literature provides limited data on the effects of CytoSorb^®^ adsorption on this selected population of patients [3,4,5], which is why we found it important to highlight the peculiar aspects of our results.

Thus far, most studies have focused on the effects of CytoSorb^®^ in patients undergoing cardiac surgery without liver failure [6,7,8] or in critically ill patients with septic shock [9,10]. Notably, in two studies, despite the reported indication being ‘liver indication’ [11] or ‘acute liver dysfunction’ [12], the patients were critically ill individuals with multiple organ failure that included liver involvement rather than those with primary liver failure. This distinction is crucial, as it suggests that the liver dysfunction in these patients may be part of a more complex syndrome of critical illness, which could complicate the evaluation of treatment outcomes. Regarding the duration of individual treatments, we adhered to the manufacturer’s instructions, setting the maximum duration of a single treatment at 24 h. This approach was implemented to ensure optimal efficacy while maintaining patient safety during the adsorption process.

Regarding the number of treatments, our decision to perform two or three sessions per week was influenced by several factors. These included the treatment frequency reported in various studies in the literature, which typically ranges from two to three treatments, as well as the progression in each individual case. Key considerations were the availability of an organ for transplantation, improvements in bilirubin trends, and death. Additionally, we carefully monitored for any side effects that could have potentially elevated the risk of death to an unsustainable level.

Finally, regarding the purifying efficacy of each treatment in terms of removing albumin-related molecules, our study confirmed that CytoSorb^®^ was effective in significantly reducing total bilirubin levels.

In response to our colleagues’ request, we have attached a new table/figure displaying the total bilirubin values recorded before and after each CytoSorb^®^ session (Figure 1). These data provide clearer insight into the treatment outcomes and reinforce the concerns about the therapy’s effectiveness in managing very high bilirubin levels.

Notably, based on these intermediate measurements, we decided to stop treatment even before the established 24 h if we observed only a very modest reduction in bilirubin between two adjacent time points. In such cases, we inferred that the CytoSorb^®^ adsorption capacity was likely decreasing, which prompted us to discontinue the treatment to avoid unnecessary prolongation without significant therapeutic benefit.

We concur with the authors’ suggestion that incorporating mass balance calculations could enhance the evaluation of the purification system’s effectiveness and help determine the optimal timing for discontinuing treatment [13]. This aspect could certainly be explored in future research.

Interestingly, mass balance calculations have so far only been performed in one clinical study [13] and one in vitro study [14] and have not yet been implemented in clinical practice. Notably, the clinical study was published after the submission of our retrospective study [2].

This indicates a significant gap in applying these methodologies in real-world scenarios, highlighting the need for further investigation in clinical settings.

Furthermore, only a limited number of studies have evaluated the increase in bilirubin levels 24 to 72 h after the discontinuation of CytoSorb^®^ treatment [13,15].

In a recent meta-analysis that selected 33 publications for a total number of 323, the comparison in pooled cases between pre-and post-treatment blood levels showed significantly reduced AST and vasopressor need but a non-significant reduction in total bilirubin levels, ALT, C-reactive protein, and creatinine [12]. In this large study, the effect on the platelets was not investigated. Notably, none of the selected studies reported the success rate or any other descriptive outcomes about bridging to liver transplantation [12]. In our observations, we noted an increase in bilirubin levels in four out of seven patients. Importantly, we do not attribute this rise to the release of bilirubin from the sorbent, which is well regarded for its capacity to bind bilirubin effectively. Instead, we believe this increase is more indicative of the progressive worsening of liver damage in these patients.

Cirrhotic patients, before resective or transplant surgery, suffer from thrombocytopenia [16]. Thrombocytopenia is among the determinants of high transfusion needs during liver surgery [17]. This finding is even more evident during the early postoperative days, particularly in patients with severe liver dysfunction [18,19]. Notably, two prognostic scores included low platelets as a poor prognostic determinant [20].

The reduction in platelet count appeared to be a significant side effect, particularly given that most of our studied population exhibited very low platelet counts to begin with.

While others have reported similar findings [3,4,21], the levels we observed are concerning and do not permit the application of any form of extracorporeal treatment. This raises important questions regarding the safety and feasibility of such interventions in this patient population.

In addition, we believe that the use of CytoSorb^®^ should be carefully evaluated in patients with liver failure who do not also have renal failure, as the application of the extracorporeal circuit can further exacerbate the reduction in platelet count. Patients with liver failure, particularly those experiencing liver dysfunction after transplantation, often present with very low platelet counts; the concurrent use of CytoSorb^®^ alongside continuous renal replacement therapy (CRRT) can significantly worsen thrombocytopenia. This issue is not commonly observed in other patients treated with CytoSorb^®^.

Regarding D-dimers, we were among the few to report these data, even if this issue warrants further investigation. To date, most studies on D-dimer levels have focused on patients with COVID-19 treated with CytoSorb^®^ [22,23], while data concerning liver patients remain fairly limited [15]. This gap highlights the need for additional research to better understand the implications of D-dimer levels in this specific population and how they may relate to the use of CytoSorb^®^ in the management of liver dysfunction.

In summary, we conclude that CytoSorb^®^ is useful in the management of a relevant number of patients with hyperbilirubinemia. Careful consideration must be given before initiating treatment in patients with liver failure and thrombocytopenia, particularly those with cirrhosis, hypersplenism, or an early postoperative course after liver transplantation or major liver resection. We suggest mitigating the risk of hemorrhagic complications by platelet transfusion. Notably, since some of these patients also require kidney depurative treatment, which can be performed exclusively by extracorporeal dialysis, we do suggest the association of CytoSorb^®^ for these patients. Further multicenter randomized trials are needed to examine the effects of CytoSorb^®^ treatment on the platelet and coagulation profiles of these patients and to evaluate the effect on the outcome.

## Figures and Tables

**Figure 1:**
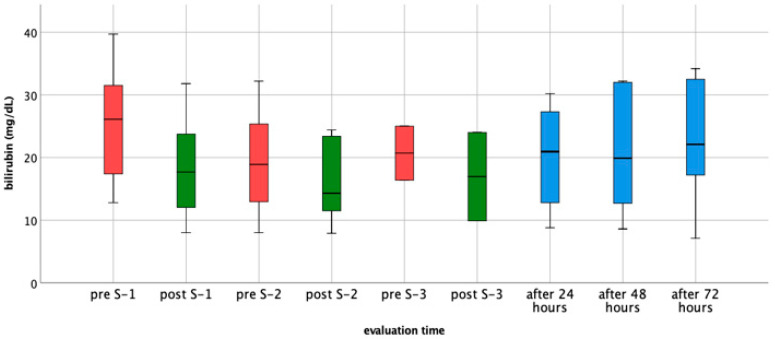
Trend of total bilirubin levels (mg/dL) at 9 evaluation times. Pre-S1 (before session #1, available in 7 cases), post-S1 (after session #1, available in 7 cases), pre-S2 (before session #2, available in 7 cases), post-S2 (after session #2, available in 7 cases), pre-S3 (before session #3, available in 2 cases), post-S3 (after session #3, available in 2 cases), after 24h (after 24 h, available in 6 cases), after 48 h (after 48 h, available in 5 cases), and after 72 h (after 72 h, available in 5 cases).
